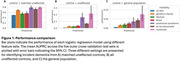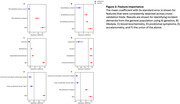# Predicting incident dementia in the UK Biobank with smartwatches

**DOI:** 10.1002/alz.095464

**Published:** 2025-01-09

**Authors:** Ann‐Kathrin Schalkamp, Kathryn J Peall, Neil A Harrison, Valentina Escott‐Price, Payam Barnaghi, Cynthia Sandor

**Affiliations:** ^1^ Imperial College London, London United Kingdom; ^2^ UK Dementia Research Institute, Care Research and Technology Centre, London United Kingdom; ^3^ Neuroscience and Mental Health Innovation Institute, Cardiff United Kingdom; ^4^ Cardiff University Brain Research Imaging Centre (CUBRIC), Cardiff United Kingdom; ^5^ UK Dementia Research Institute, Cardiff University, Cardiff United Kingdom; ^6^ Cardiff University, Cardiff United Kingdom; ^7^ Great Ormond Street Hospital NHS Foundation Trust, London United Kingdom; ^8^ UK Dementia Research Institute, Imperial College London, London United Kingdom

## Abstract

**Background:**

The early detection of dementia is paramount for clinical study design and early treatment. Smartwatches allow for passive data collection of behavior, making it an accessible and scalable data source. Previous studies have demonstrated the efficacy of smartwatch data in identifying incident Parkinson’s disease. This study seeks to explore the potential of smartwatch‐derived data in predicting incident dementia.

**Method:**

Using UK Biobank, we investigated the predictive value of accelerometry in identifying incident (N = 201) all cause dementia in the general population (N = 33009), including healthy controls and other diseases). We further examined performance in identification from age and sex matched healthy control (N = 201) and from all healthy control (N = 24987). We compared the performance with models based on genetics, lifestyle, blood biochemistry or prodromal symptoms data. Feature analysis identified the most stable contributors to the risk prediction.

**Result:**

A machine learning model trained using accelerometry data achieved higher test performance (Figure 1) for identifying incident dementia in the general population (AUPRC 0.04±0.01, prevalence: 0.006) than models based on genetics (p‐value = 0.008) and lifestyle (p‐value = 0.02). It performed on‐par with models based on blood biochemisty (AUPRC 0.03±0.005) and prodromal symptoms (AUPRC 0.04±0.02). A combined model achieved highest performance (AUPRC 0.08±0.03) with identified important and stable features (Figure 2) including age (mean coefficient and standard error: 0.93±0.08), polygenic risk score for Alzheimer’s disease (0.53±0.06), mean acceleration during light physical activity (‐0.29±0.04), and maximum time spent continuously asleep (‐0.36±0.03).

**Conclusion:**

Accelerometry with features describing activity and sleep did not outperform other modalities in the identification of incident dementia. Further features derived from accelerometry need to be investigated for their potential use in early screening for dementia.